# Characterization of conserved properties of hemagglutinin of H5N1 and human influenza viruses: possible consequences for therapy and infection control

**DOI:** 10.1186/1472-6807-9-21

**Published:** 2009-04-07

**Authors:** Veljko Veljkovic, Nevena Veljkovic, Claude P Muller, Sybille Müller, Sanja Glisic, Vladimir Perovic, Heinz Köhler

**Affiliations:** 1 Center for Multidisciplinary Research, Institute of Nuclear Sciences VINCA, P.O. Box 522, 11001 Belgrade, Serbia; 2 Institute of Immunology and WHO Collaborative Center for Measles and WHO European Regional Reference Laboratory for Measles and Rubella, Laboratoire National de Santé Luxembourg, 20A rue Auguste Lumière, L-1950 Luxembourg, Grand-Duchy of Luxembourg; 3 Immpheron Inc., 5235 Athens-Boonesboro Rd., Lexington, Kentucky 40509, USA; 4 Department of Microbiology and Immunology, University of Kentucky, Markey Cancer Center, Combs 203, 800 Rose Street, Lexington, Kentucky 40536, USA

## Abstract

**Background:**

Epidemics caused by highly pathogenic avian influenza virus (HPAIV) are a continuing threat to human health and to the world's economy. The development of approaches, which help to understand the significance of structural changes resulting from the alarming mutational propensity for human-to-human transmission of HPAIV, is of particularly interest. Here we compare informational and structural properties of the hemagglutinin (HA) of H5N1 virus and human influenza virus subtypes, which are important for the receptor/virus interaction.

**Results:**

Presented results revealed that HA proteins encode highly conserved information that differ between influenza virus subtypes H5N1, H1N1, H3N2, H7N7 and defined an HA domain which may modulate interaction with receptor. We also found that about one third of H5N1 viruses which are isolated during the 2006/07 influenza outbreak in Egypt possibly evolve towards receptor usage similar to that of seasonal H1N1.

**Conclusion:**

The presented results may help to better understand the interaction of influenza virus with its receptor(s) and to identify new therapeutic targets for drug development.

## Background

Influenza is currently considered as one of the most severe threats to human health and animal welfare. The highly pathogenic avian influenza (HPAIV) H5N1 viruses have been isolated from avian species in more than 50 countries. As of January 2008, 349 human H5N1 infections have been reported to the World Health Organization (WHO) [1]. Of these 349 cases, 216 patients have died (62%) and there has been no decline in mortality rate. Because the virus has evolving antigenicity for which humans may not have a pre-existing immunity, the conditions for a possible pandemic exist.

The entry of influenza virus into susceptible cells is mediated by the viral hemagglutinin (HA) membrane glycoprotein which binds sialic acids of cell-surface glycoproteins and glycolipids. The binding preference of a given HA for different receptors correlates to some extent with the species specificity for infection. Human isolates preferentially bind to receptors with α2,6 linkages to galactose (SAα2,6Gal), whereas avian isolates prefer α2,3 linkages (SAα2,3Gal) [2-6]. A change in receptor preference is, however not necessary since the lower respiratory tract also expresses α 2.3 receptors [7]. It has also been reported that influenza virus can infect host cells via a sialic acid-independent pathway, either directly or in a multistage process [8]. It has been speculated that sialic acid enhances virus binding to secondary receptors that mediate entry [8].

Several approaches, such as structural analyses, model protein evolution, and mathematical modeling have been taken to study the antigenic drift and shift of influenza A viruses (for review see Ref. [23] and references therein). All of these approaches trace changes in HA but they do not allow precise assessment of biological consequences. Here we applied the informational spectrum method (ISM), which is a theoretical approach to investigate the periodicity of structural motifs with defined physicochemical characteristics that determinate biological properties of proteins [9]. The protein sequence is encoded numerically by assigning to each amino acid its electron-ion interaction potential (EIIP), which describes the average energy states of valence electrons in an amino acid. By using the discrete Fourier transform (DFT), the numerical sequence is transformed into a frequency domain to create an ISM spectrum. It has been pointed out that the Fourier spectra of protein sequences involved in mutual interaction are similar and this similarity is represented by the common frequency component [9]. The ISM spectrum is a contribution of all individual amino acids in the sequence. Therefore, once the characteristic frequency has been identified it is possible to use ISM to determine how the substitution of an amino acid changes the frequency and influences the biological activity of the protein. Using this bioinformatics approach we have previously characterized the conserved information responsible for interaction between envelope glycoprotein gp120 of human immunodeficiency virus type 1 (HIV-1) and their CD4, CCR5 and CXCR4 receptors [9-11]. By analogy with HIV-1 gp120, it can be assumed that highly variable HA molecules of influenza viruses also encode conserved information, which may determine receptor-binding preferences. Identification and characterization of this information could contribute to a better understanding of HPAIV/host interaction.

Here we show that the HA subunit 1 (HA1) of H5N1 viruses encodes specific and highly conserved information which may determine the recognition and targeting of these HPAI viruses to their receptor. The comparison with seasonal strains suggests that a subset of H5N1 in Egypt may be evolving towards an H1N1-like receptor usage.

## Methods

### Sequences

**Figure 1:**
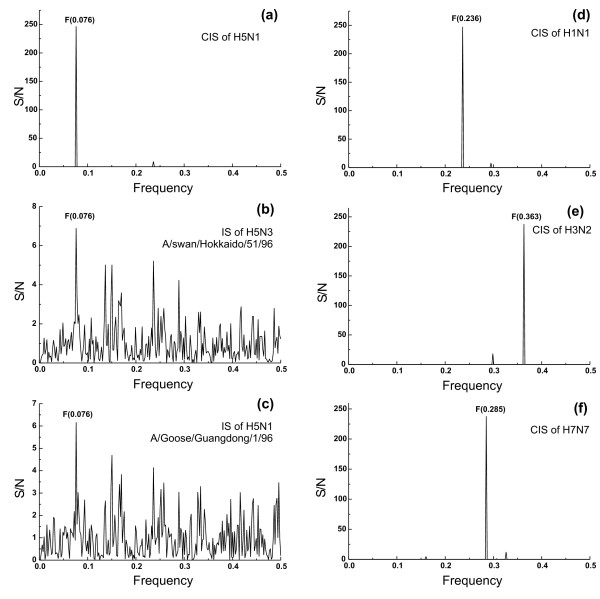
**ISM analysis of HA1 proteins of H5 influenza viruses**. (a) Consensus IS of HA1 of all H5N1 sequences in GenBank (n = 1407); (b) IS of H5N3 (A/swan/Hokkaido/51/96), the progenitor of H5N1, and (c) of the first isolated H5N1 virus (A/Goose/Guangdong/1/96); (d) consensus IS of H1N1 (n = 30), (e) of H3N2 (n = 30) and (f) of H7N7 (n = 30).

**Figure 2:**
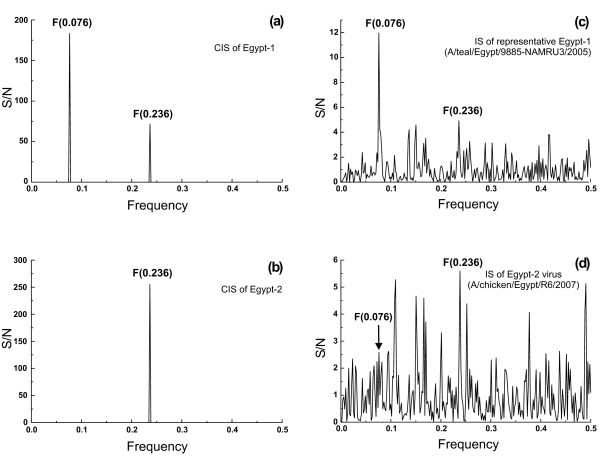
**The ISM analysis of HA1 proteins of H5N1 viruses isolated in Egypt in 2006/2007**. (a) consensus IS of Egypt-1 and (b) of Egypt-2 strains, (c) IS of a representative Egypt-1 (A/teal/Egypt/9885-NAMRU3/2005) and (d) Egypt-2 virus (A/chicken/Egypt/R6/2007).

The HA1 sequences were retrieved from GenBank database with following accession numbers and were used for the results of Figure 1 and 2:

**H1N1 **(Figure 1d): A/Fort Monmouth/1/1947-mouse adapted [U02464]; A/Fort Monmouth/1/1947 [U02085]; A/Lepine/1948 [AB043479]; A/TF/15/1951 [AB043480]; A/Kojiya/1/1952 [AB043482]; A/Finland/158/1991 [L19549]; A/Taiwan/13/1954 [AB043483]; A/Meguro/1/1956 [AB043485]; A/Saga/2/1957 [AB043486]; A/swine/Wisconsin/1/1968 [EU139825]; A/swine/Iowa/1973 [EU139826]; A/swine/Iowa/1976/1931 [U11858]; A/chicken/Hong Kong/14/1976 [EF679200]; A/USSR/90/1977 [DQ508897]; A/duck/Hong Kong/717/1979 [EF679199]; A/Kiev/59/1979 [M38353]; A/turkey/Kansas/4880/1980 [EF679201]; A/swine/Netherlands/3/1980 [U46942]; A/swine/Netherlands/12/1985 [U46943]; A/Singapore/6/1986 [D00406]; A/Taiwan/01/1986 [DQ508873]; A/Kamata/85/1987 [AB043487]; A/SL/2/1987 [M33748]; A/Fiji/2/1988 [L19011]; A/South Carolina/6/1988 [L19025]; A/Franch/6908/1989 [L19016]; A/Yamagata/32/1989 [AB304820]; A/Stockholm/26/1990 [L19013]

**H3N2 **(Figure 1e): A/Aichi/2/1968 [EF614248]; A/England/878/1969 [K03335]; A/Qu/7/1970 [K03338]; A/Hong Kong/107/1971 [EF626615]; A/Udorn/307/1972 [DQ508929]; A/Port Chalmers/1/1973 [EF626618]; A/Victoria/3/1975 [EF626609]; A/duck/Alberta/78/1976 [M73771]; A/swine/Italy/1850/1977 [DQ975252]; A/swine/Hong Kong/81/1978 [M19057]; A/Bangkok/01/1979 [DQ508825]; A/Shanghai/31/1980 [EF626620]; A/swine/Italy/6/1981 [DQ975253]; A/Umea/1982/92 [AY661134]; A/swine/Ukkel/1/1984 [M73775]; A/Leningrad/360/1986 [DQ508849]; A/swine/Italy/630/1987 [DQ975255]; A/Hokkaido/1/1988 [D43787]; A/Beijing/352/1989 [D43786]; A/swine/Ange-Gardien/150/1990 [U07146]; A/Wisconsin/03/2007 [EU516105]; A/Hokkaido/1/1993 [D43788]; A/England/79/1994 [EF456783]; A/swine/Italy/1380-2/1995 [DQ975260]; A/Lyon/1781/96 [AF131996]; A/Nairobi/2041/2006 [EF199897]; A/Panama/2007/1999 [DQ508865]; A/South Africa/96/2000 [EF462562]; A/Chile/6416/2001 [DQ865972]

**H5N1 **(Figure 1a): All available H5N1 HA1 sequences presented in the GenBank database (1407 entries) as of 1 February 2008.

**H5N1 (Egypt-1) **(Figure 2a): A/Egypt/2763-NAMRU3/2006 [EF042614]; A/Egypt/2782-NAMRU3/2006 [DQ464377]; A/Egypt/12374-NAMRU3/2006 [EF061116]; A/Chicken/Egypt/5611NAMRU3-AN/2006 [DQ837588]; A/Chicken/Egypt/5610NAMRU3-F3/2006 [DQ837587]; A/Turkey/Egypt/5613NAMRU3-T/2006 [DQ837590]; A/Chicken/Egypt/5612NAMRU3-S/2006 [DQ837589]; A/chicken/Egypt/960N3-004/2006 [DQ447199]; A/chicken/Egypt/10845-NAMRU3/2006 [EF042622]; A/chicken/Egypt/2253-1/2006 [DQ862001]; A/turkey/Egypt/2253-2/2006 [CY020653]; A/chicken/Egypt/1300-NAMRU3/2007 [EF441280]; A/chicken/Egypt/1078-NAMRU3/2006 [EF441276]; A/chicken/Egypt/1890N3-HK45/2007 [EF469654]; A/chicken/Egypt/1891N3-CLEVB/2007 [EF469659]; A virus (A/Egypt/1902-NAMRU3/2007 [EF535820]; A/Egypt/5614-NAMRU3/2006 [EF042621]; A/Egypt/2256-NAMRU3/2007 [EF535821]; A/Egypt/2321-NAMRU3/2007 [EF535822]; A/Egypt/2331-NAMRU3/2007 [EF535823]; A/Egypt/2616-NAMRU3/2007 [EF535824]; A/Egypt/2620-NAMRU3/2007 [EF535825]; A/Egypt/3458-NAMRU3/2006 [EF042619]; A/chicken/Egypt/R1/2006 [EU183327]; A/duck/Egypt/F5/2006 [EU183325]; A/chicken/Egypt/F4/2006 [EU183324]; A/chicken/Egypt/F3/2006 [EU183323]; A/turkey/Egypt/F2/2006 [EU183322]; A/Egypt/5494-NAMRU3/2006 [EF042620]; A/Egypt/3105-NAMRU3/2006 [EF042618]; A/Egypt/2630-NAMRU3/2007 [EU095026]; A/Egypt/2750-NAMRU3/2007 [EU095028]; A/Egypt/2751-NAMRU3/2007 [EU095029]; A/Egypt/4082-NAMRU3/2007 [EU095031]; A/Egypt/4226-NAMRU3/2007 [EU095032]; A/Egypt/6251-NAMRU3/2007 [EU095033]; A/Egypt/4081-NAMRU3/2007 [EU095030]; A/Egypt/2786-NAMRU3/2006 [EF042616]; A/Egypt/2783-NAMRU3/2006 [EF042615]; A/chicken/Egypt/07181-NLQP/2007 [EU496387]; A/chicken/Egypt/07201-NLQP/2007 [EU496388]; A/chicken/Egypt/07202-NLQP/2007 [EU496389]; A/turkey/Egypt/07203-NLQP/2007 [EU496390]; A/duck/Egypt/07264S-NLQP/2007 [EU496391]; A/goose/Egypt/07364S-NLQP/2007 [EU496393]; A/turkey/Egypt/07444S-NLQP/2007 [EU496394]; A/chicken/Egypt/07632S-NLQP/2007 [EU496395]; A/chicken/Egypt/07665S-NLQP/2007 [EU496396]; A/chicken/Egypt/07701S-NLQP/2007 [EU496397]; A/chicken/Egypt/06553-NLQP/2006 [EU496383]; A/chicken/Egypt/06612-NLQP/2006 [EU496384]; A/quail/Egypt/07120-NLQP/2007 [EU496385]; A/chicken/Egypt/07125-NLQP/2007 [EU496386]

**Figure 3:**
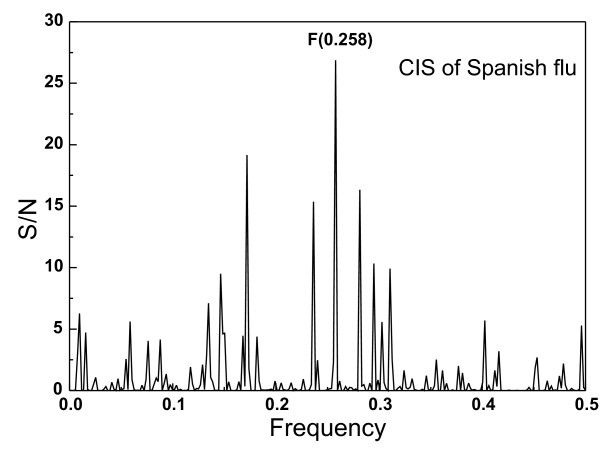
**Consensus IS of HA1 from three Spanish flu H1N1 viruses**.

**H5N1 (Egypt-2) **(Figure 2b): A/Egypt/0636-NAMRU3/2007 [EF382359]; A/Egypt/14725-NAMRU3/2006 [EF200513]; A/duck/Egypt/2253-3/2006 [DQ862002]; A/duck/Egypt/1301-NAMRU3/2007 [EF441281]; A/chicken/Egypt/1081-NAMRU3/2006 [EF441279]; A/chicken/Egypt/1080-NAMRU3/2006 [EF441278]; A/chicken/Egypt/1079-NAMRU3/2007 [EF441277]; A/Egypt/14724-NAMRU3/2006 [EF200512]; A/chicken/Egypt/1889N3-SM26/2007 [EF469653]; A/chicken/Egypt/1892N3-HK49/2007 [EF469660]; A/duck/Egypt/1888N3-SM25/2007 [EF469657]; A/Egypt/1394-NAMRU3/2007 [EF535817]; A/Egypt/1604-NAMRU3/2007 [EF535818]; A/Egypt/1731-NAMRU3/2007 [EF535819]; A/Egypt/2621-NAMRU3/2007 [EF535826]; A/goose/Egypt/R4/2007 [EU183330]; A/chicken/Egypt/F6/2007 [EU183326]; A/chicken/Egypt/R6/2007 [EU183332]; A/duck/Egypt/R5/2007 [EU183331]; A/chicken/Egypt/R3/2007 [EU183329]; A/chicken/Egypt/R2/2007 [EU183328]; A/Egypt/2629-NAMRU3/2007 [EU095025]; A/Egypt/2631-NAMRU3/2007 [EU095027]; A/Egypt/2947-NAMRU3/2006 [EF042617]; A/chicken/Egypt/9385NAMRU3-CLEVB125/2007 [EU371910]; A/chicken/Egypt/9390NAMRU3-CLEVB157/2007 [EU371915]; A/chicken/Egypt/9392NAMRU3-CLEVB167/2007 [EU371917]; A/chicken/Egypt/9387NAMRU3-CLEVB148/2007 [EU371912]; A/chicken/Egypt/3051NAMRU3-CLEVB78/2007 [EU371905]; A/chicken/Egypt/9386NAMRU3-CLEVB/136/2007 [EU371911]; A/chicken/Egypt/9391NAMRU3-CLEVB158/2007 [EU371916];

**H7N7 **(Figure 1f): A/chicken/Victoria/75 [Z47199]; A/tern/Potsdam/342/6/79 [U20470]; A/chicken/Jena/1816/87 [U20469]; A/swan/Potsdam/63/6/81 [U20467]; A/duck/Heinersdorf/S495/6/86 [U20465]; A/equine/London/1416/1973 [M58657]; A/starling/Victoria/1/1985 [M17736]; A/goose/Leipzig/137/8/1979 [L43913]; A/seal/Mass/1/80 [K00429]; A/chicken/Netherlands/03010132/03 [EF015551]; A/mallard/Italy/4810-7/2004 [DQ838514]; A/Mallard/Sweden/107/02 [AY999991]; A/equine/Santiago/77 [AY383756]; A/chicken/Netherlands/1/03 [AY338458]; A/chicken/Germany/R28/03 [AJ620350]; A/turkey/Ireland/PV8/95 [AJ704799]; A/ostrich/South Africa/M320/96 [AF202253]; A/macaw/England/626/80 [AF202250]; A/chicken/Ireland/1733/89 [AF202239]; A/non-psittacine/England-Q/1985/89 [AF202240]; A/turkey/Ireland/PV74/1995 [AF028021]; A/England/268/1996 [AF028020]; A/duck/Jiangxi/1814/03 [EU158103]; A/duck/Jiangxi/1742/03 [EU158108]; A/duck/Jiangxi/1786/03 [EU158102]; A/mallard/Italy/299/05 [EU158104]; A/duck/Jiangxi/1760/03 [EU158101];

**H1N1 **(Spanish flu) (Figure 3): A/South Carolina/1/18 [AF117241]; A/New_York/1/18 [AF116576]; A/Brevig_Mission/1/18 [AF116575]

### Informational spectrum method

The surface complementarity between interacting biomolecules, which was originally proposed by Emil Fischer in 1894, together with the collision theory, assuming that the first contact between interacting molecules is achieved accidentally by the thermal motions that cause molecular wander, represents the fundamental basis for our current understanding of intermolecular interaction in biological systems. According to this concept, the diffusion-limited association rate constant, calculated by the Smoluchowski's equation is ~10^6 ^M^-1^s^-1 ^for a protein-ligand and ~10^3 ^M^-1^s^-1 ^for a protein-protein interaction. On the other hand, the real protein-protein association generally occurs at rates that are 10^3 ^to 10^4 ^times faster than would be predicted from a simple 3D "random diffusion" model [12].

In order to overcome the discrepancy between theoretically estimated values and real values of the associated rate constant for a protein-protein interaction, the model for interaction between biological molecules based on frequency-selective long-range attractive forces which are efficient at a distance longer than one linear dimension of the interacting macromolecules (10^2 ^– 10^3 ^Å), has been proposed [13,14]. It has been shown that the number of valence electrons and EIIP, representing the main energy term of the valence electrons, are essential physical parameters of biological molecules determining their long-range properties of biological molecules. The EIIP can be determined for organic molecules by the following simple equation derived from the "general model pseudopotential" [15,16]:

(1)

where Z* is the average quasivalence number (AQVN) determined by

(2)

where Z_i _is the valence number of the i-th atomic component, n_i _is the number of atoms of the i-th component, m is the number of atomic components in the molecule, and N is the total number of atoms. The EIIP values calculated according to equations (1) and (2) are in Rydbergs (Ry).

Using the concept of the long-range forces which increase numbers of productive collisions between interacting biomolecules and the EIIP values of amino acids, the informational spectrum method (ISM), for analysis of protein-protein interaction and the relationship between structure and function of proteins, was developed. This virtual spectroscopy method comprises three basic steps:

Transformation of the alphabetic code of the primary structure into a sequence of numbers by assigning to each amino acid or nucleotide a corresponding numerical value representing the electron-ion interaction potential.

Conversion of the obtained numerical sequence by Fourier transformation into the informational spectrum (IS).

Cross-spectral analysis which allows identification of frequency components in the informational spectrum of molecules which are important for their biological function or interaction with other molecules.

The physical and mathematical basis of ISM was described in detail elsewhere [17-20], and here we will only present this bioinformatics method in brief. A sequence of N residues is represented as a linear array of N terms, with each term given a weight. The weight assigned to a residue is EIIP (Table 1). In this way the alphabetic code is transformed into a sequence of numbers. The obtained numerical sequence, representing the primary structure of protein, is then subjected to a DFT, which is defined as follows:

**Table 1:** The electron- ion interaction potential (EIIP) of amino acids used to encode amino acids.

**Amino acid**	**EIIP [Ry]**
Leu	0.0000

Ile	0.0000

Asn	0.0036

Gly	0.0050

Glu	0.0057

Val	0.0058

Pro	0.0198

His	0.0242

Lys	0.0371

Ala	0.0373

Tyr	0.0516

Trp	0.0548

Gln	0.0761

Met	0.0823

Ser	0.0829

Cys	0.0829

Thr	0.0941

Phe	0.0946

Arg	0.0959

Asp	0.1263

(3)

where x(m) is the m-th member of a given numerical series, N is the total number of points in this series, and X(n) are DFT coefficients. These coefficients describe the amplitude, phase and frequency of sinusoids, which comprise the original signal. The absolute value of complex DFT defines the amplitude spectrum and the phase spectrum. The complete information about the original sequence is contained in both spectral functions. However, in the case of protein analysis, relevant information is presented in an energy density spectrum [17,18], which is defined as follows:

(4)

In this way, sequences are analyzed as discrete signals. It is assumed that their points are equidistant with the distance d = 1. The maximal frequency in a spectrum defined in this way is F = 1/2d = 0.5. The frequency range is independent of the total number of points in the sequence. The total number of points in a sequence influences only the resolution of the spectrum. The resolution of the N-point sequence is 1/n. The n-th point in the spectral function corresponds to a frequency f(n) = nf = n/N. Thus, the initial information defined by the sequence of amino acids can now be presented in the form of IS, representing a series of frequencies and their amplitudes.

The IS frequencies correspond to distribution of structural motifs with defined physicochemical properties determining a biological function of a protein. When comparing proteins, which share the same biological or biochemical function, the ISM technique allows detection of code/frequency pairs which are specific for their common biological properties, or which correlate with their specific interaction. This common informational characteristic of sequences is determined by a cross-spectrum or consensus informational spectrum (consensus IS). A consensus IS of N spectra is obtained by the following equation:

(5)

where Π (i, j) is the j-th element of the i-th power spectrum and C(j) is the j-th element of consensus IS. Thus, consensus IS is the Fourier transform of the correlation function for the spectrum. In this way, any spectral component (frequency) not present in all compared IS is eliminated. Peak frequencies in consensus IS are common frequency components for the analyzed sequences. A measure of similarity for each peak is a signal-to-noise ratio (S/N), which represents a ratio between signal intensity at one particular IS frequency and the main value of the whole spectrum. If one calculates a consensus IS for a group of proteins, which have different primary structures, and finds strictly defined peak frequencies, it means that the analyzed proteins participate in mutual interaction or have a common biological function.

The ISM was successfully applied in structure-function analysis of different protein sequences and *de novo *design of biologically active peptides (for review see Refs. 10 and 20), assessment of biological effects of mutations [21] and prediction of new protein interactors [22].

## Results

**Figure 4:**
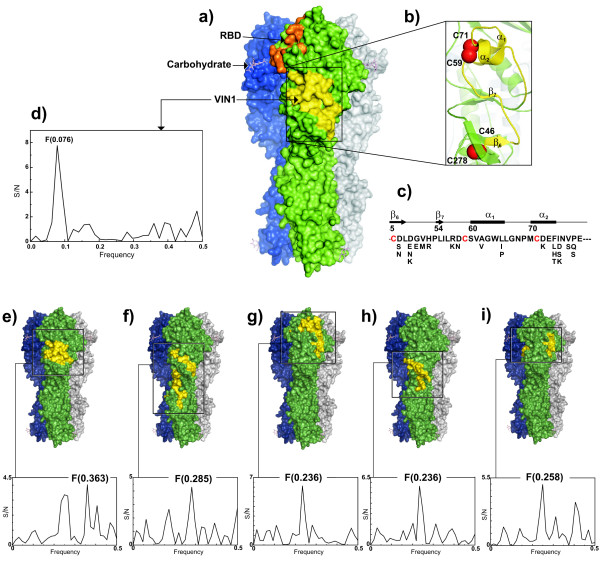
**Overview of H5 HA trimer **(PDB: 2ibx)** and details of the VIN1 region**. (a) Surface of HA trimer; each monomer has a different color. Carbohydrates are orange. The location of receptor binding domain (orange) and VIN1 region (yellow) are highlighted only for one monomer. (b) Ribbon representation of VIN1 region (yellow). Sulphur atoms involved in stabilization of the VIN1 region are shown as red spheres. Figures were generated by PyMol. (c) Secondary structure and amino acid composition of H5 HIN1 region. The consensus sequence of the VIN1 region is shown together with mutations found in 595 H5 HA sequences using BioEdit. Cystein residues are shown in red. (d) IS of the VIN1 region. Domains of H1N1, H3N2, H5N1, H7N1 and Spanish flu identified by consensus IS (Table 2) and their position in the 3D structure of HA1 and the IS of the peptide sequence. (e) A/New York/383/2004 (H3N2); (f) A/equine/Prague/56 (H7N7); (g) A/Egypt/0636-NAMRU3/2007(H5N1); (h) A/New Caledonia/20/99 (H1N1); (i) A/South Carolina/1/18 (H1N1).

To identify conserved information encoded by HA1 proteins, we performed a cross-spectral analysis of all H5N1 HA1 amino acid sequences in GenBank (1407 entries). Figure 1a shows that the consensus IS of these sequences contains only one peak of the frequency F(0.076). According to the ISM concept, this information represents the long-range component of the protein-protein interaction between HA1 and a putative partner, such as a receptor. Figures 1b and 1c show the IS of HA1 of the H5N3 virus A/swan/Hokkaido/51/96, the putative progenitor of the HPA1 H5N1 subtype, and of the first H5N1 virus isolated in China 2006 (A/Goose/Guangdong/1/96) [24]. Both of these IS have a dominant peak at the same characteristic frequency F(0.076), demonstrating that HA of these two viruses encode the same information as the H5N1 HA1 shown in Figure 1a. The computer scanning survey of the primary structure of H5N1 HA1 showed that the main contribution to information represented by the frequency F(0.076) comes from the domain (denoted VIN1) located in the N-terminus of the protein which encompasses residues 42 – 75 of the mature protein (Table 2, Figure 4). Interestingly, this domain of H5N1 HA1 is highly conserved in all H5N1 viruses.

**Table 2:** The receptor recognition domains of HA proteins from H5N1, H1N1, H3N2 and H7N7 influenza viruses.

**Strain**	**Frequency**	**Residues**	**Sequence**
A/Hong Kong/213/03 (H5N1)	F(0.076)	42 – 75	CDLDGVKPLILRDCSVAGWLLGNPMCDEFINVPE

A/New Caledonia/20/99 (H1N1)	F(0.236)	262 – 295	SGIITSNAPMDECDAKCQTPQGAINSSLPFQNVH

A/New York/383/2004 (H3N2)	F(0.363)	57 – 90	QILDGENCTLIDALLGDPQCDGFQNKKWDLFVER

A/equine/Prague/56 (H7N7)	F(0.285)	28 – 61	GIEVVNATETVEQTNIPKICSKGKQTVDLGQCGL

A/Egypt/0636-NAMRU3/2007(H5N1)	F(0.236)	99 – 132	EELKHLLSRINHFEKIQIIPKNSWSDHEASGVSS

A/South Carolina/1/18 (H1N1))	F(0.258)	87 – 120	NSENGTCYPGDFIDYEELREQLSSVSSFEKFEIF

Next, we performed the ISM analysis of HA1 molecules of seasonal viruses H1N1 (n = 29) and H3N2 (n = 30), as well as H7N7 viruses (n = 30), from different years and geographic regions. Their consensus IS show characteristic peaks of the frequencies F(0.236), F(0.363) and F(0.285), respectively (Figures. 1d, e and 1f), distinct from the F(0.076) of H5N1 HA. This may suggest that HA1 sequences encode information which is specific for each of these subtypes. The domains of HA1 of H1N1, H3N2, H7N7 influenza viruses, derived from the above frequencies are shown in Table 2 and highlighted in the HA structural model (Figure 4).

Despite its low infectivity for humans, there has been evidence in Egypt of several clusters of human-to-human transmission with very high mortality rate. ISM analysis of 95 HA sequences from Egypt 2006 and 2007 showed that these viruses can be divided into two groups. Consensus IS of a first group (Egypt-1) of 55 strains contains a dominant peak of the frequency F(0.076) which is characteristic for H5N1 HA1 and a less prominent peak of the frequency F(0.236) which is characteristic for H1N1 HA1 (Figure 2a). In contrast, consensus IS of the second group (Egypt-2) (Figure 2b), which includes 40 H5N1 HA1, contains only one significant peak of the frequency F(0.236) corresponding to the consensus IS of H1N1 HA1 in Figure 1d. Figures 2c and 2d show representative IS of individual strains of both groups. Of H5N1 viruses which were isolated in Egypt during 2006, 76% belong to the group Egypt-1, and 24% were from the group Egypt-2. In contrast, in 2007, 48% belong to the Egypt-1 and 52% to Egypt-2.

Figure 4 shows the IS spectra of peptide VIN1 and of the domains identified by consensus IS of H1N1, H3N2, H5N1 and H7N7 viruses (Table 2) and the position of these domains in the molecule. As can be seen, the receptor targeting site of H5N1 virus from the group Egypt-1 (A/Egypt/0636-NAMRU3/2007) is closer to the receptor binding site than in the other viruses of Figure 4. It may be speculated that this may affect the efficacy of the virus/receptor interaction.

Finally, we compared informational properties of H1N1 pandemic strains from 1918 from GenBank and seasonal H1N1 strains. The consensus IS of these pandemic isolates (Figure 3) is characterized by a dominant peak of the frequency F(0.258) which is different from the frequency F(0.236) characteristic of other seasonal flu H1N1 isolates (Figure 1d). Table 2 shows the domain corresponding to the frequency F(0.258). In the model of A/South Carolina/1/18 (Figure 4i) the position of this domain does not overlap with the corresponding domain of other seasonal H1N1 strains, but overlaps with the corresponding domain of Egypt-2 H5N1 viruses.

## Discussion

The differentiation of H5N1 in an increasing number of clades and subclades is alarming but the fundamental changes associated with efficient human to human transmission are poorly understood. The development of approaches which allow the tracing and the understanding of such changes is of the highest priority.

To identify specific information which determines long-range components of protein-protein interactions between H5N1 and putatively its receptor(s), we performed the ISM analysis of the HA1 protein. This analysis revealed that this protein, although highly variable, encodes conserved information, which is represented by the IS frequency component F(0.076). In contrast, HA1 of H1N1, H3N2 and H7N7 encode specific information reflected by different characteristic IS frequencies (F(0.236), F(0.363) and F(0.285), respectively) corresponding to different protein domains (Table 2).

The main information corresponding to the IS frequency F(0.076) is contributed by the VIN1 domain located in the N-terminus of HA1 molecule (Figure 4). This domain is highly conserved in all H5N1 viruses. The peptide VIN1 is located within the site E between residues 42 and 75, one of the five major antigenic domains of the HA molecule. In the 3D structure of HA1 the site E is located below the globular head involved in receptor binding [5]. It was previously shown that protein domains, which are essential for particular IS frequency are directly involved in protein-protein interaction [9,22]. Therefore, we postulate that the VIN1 domain plays an important role in the recognition and targeting between virus and receptor. For this reason, VIN1 may represent a potential target for therapy of H5N1 infection.

It is of note that the E site, encompassing the VIN1 domain, is placed below the globular head of HA1 which is involved in the receptor binding [5]. Most mutations which encode receptor tropism [6,7] and are involved in immune avoidance occur in this globular part of HA1 molecule. On the other hand, mutations within the site E are rare. This indicates that variable antigenic sites A and B located in the globular head of HA1 could represent an immune decoy which protects the important functional site E, determining the conserved long-range properties of the molecule. A similar structural organization was previously reported for HIV-1 gp120 [11,25] and it was pointed out as an important obstacle in development of AIDS vaccine [26-28].

H5N1 already replicates efficiently in humans, and cause case fatality rates that are ten times higher than those seen in the 1918 pandemic. Thus, an infectivity of H5N1 similar to seasonal flu would cause a catastrophic pandemic. The main obstacle for this worst case scenario is poor human-to-human transmission of H5N1 viruses, which is attributed to the paucity of sialic acid *a*2,3 receptor in the epithelium of the human upper respiratory tract, and the inability of the virus to replicate efficiently at this site. Interestingly, the ISM approach identifies important differences between H5N1 viruses from Egypt. Some have the characteristics of most H5N1 strains whereas about one third of the viruses display characteristics that are also found in human H1N1 seasonal virus. Interestingly the proportion of the latter viruses has increased from 25 to about 50: between 2006 and 2007.

Similarly the results of H5N1 strains from Egypt (Figure 2) may be indicative of a possible viral evolution towards receptor usage similar to that of H1N1 viruses, which efficiently replicate in the upper respiratory tract. The protein domain, which seems to be involved in this subtle change, corresponds to amino acid domain 99–132 (Figure 4g). However, the role of this domain for enhanced infectivity in humans remains elusive. Interestingly the corresponding domain of Spanish flu viruses and Egypt-2 H5N1 viruses are much closer to the receptor binding site of HA1 than in all other H1N1 and H5N1 viruses (Figures 4e–i and Table 2). This closer proximity may indicate more efficient virus/receptor interactions in these influenza viruses.

Finally, we will discuss some of recently reported experimental results which point out functional and immunological role of H5 HA domain encompassing peptide VIN1. In order to identify mutations which increase the recognition of H5 HA by SAα2,6Gal human type receptor, Su and co-workers compared HA from A/chicken/Ffujian/1042/2005 as wild type with isolates identified in both poultry and humans in mainland China, Hong Kong, Thailand, and Vietnam during outbreaks between 1996 and 2005 [29]. Unexpectedly, this analysis revealed six amino acid substitutions (K35R, D45N, D94N, K35R/D45N, K35R/45N/D94N, A247T) outside the receptor-binding domain of HA, which could enhance interaction between H5 HA and human-type SAα2,6Gal receptor. As can be seen, three of these mutations encompass mutation D45N which is located within peptide VIN1 and two other mutations (K35R and D94N) are located in its vicinity. It is the first report that naturally occurring mutations in region of H5 HA which encompasses peptide VIN1 play an important role in virus transmission from avian to human. It is of note that Egyptian strains contain all of these mutations, except mutation in position K35. These results point out need for future testing of evolution of Egyptian strains using hemiadsorption assays for HA receptor-binding activity in order to identify possible new mutations in this domain of HA which could increase affinity of H5N1 viruses to human-type receptor.

Du and co-workers discovered monoclonal antibody (MAb) 4G6 which efficiently and selectively recognizes and neutralizes recently emerged Asian H5N1 viruses [30]. The epitope-mapping analysis revealed that epitope of the neutralizing 4G6 MAb is located within peptide VIN1, pointing out this domain of HA as therapeutic and diagnostic target for H5N1 viruses. The 4G6 MAb recognizes residue D43 within peptide VIN1, which characterizes Asian H5N1 viruses, but not N43 which characterizes H5N2 and H5N1 viruses. It is also shown that this MAb recognizes Egyptian H5N1 strains derived from clade 2.2 containing D43. Based on these results, Du and co-workers suggested that the 4G6 MAb could be useful for rapid diagnosis of the infection of H5N1 currently circulating in Asia, Europe and Africa, as well as for development of an antibody-based therapy. It is of note that recent Egypt group-2 strains are characterized by N43, in contrast to Egypt group-1 strains which contain D43. It means that the 4G6 MAb can not be used for detection and neutralization of H5N1 viruses belonging to the Egypt group-2.

## Conclusion

In summary, the presented results showed that: (i) H5N1 HA1 encode specific information represented by an IS frequency different from that encoded by other subtypes; (ii) this characteristic frequency is largely determined by a highly conserved N-terminal domain of HA1; (iii) other subtypes encode information that corresponds to other domains including residues 262–295 for H1N1, residues 57–90 for H3N2, residues 28–61 for H7N7 and residues 87–120 for Spanish flu, (iv) at least in Egypt H5N1 viruses have acquired features that may adapt them for H1N1-like receptor usage possibly allowing more efficient human-to-human transmission. Our results suggest subtle but so far elusive differences in interactions of these different viral subtypes with their receptors. Collectively these results may help to better understand the interaction of influenza virus with its receptor(s) and to identify new targets for drug development.

## Authors' contributions

VV conceived of the study, participated in its design and coordination and preparation of the manuscript. NV carried out the ISM analysis of viral sequences. CPM performed 3D structural analysis of viral proteins and participated in preparation of the manuscript. SM contributed with immunological interpretation of results. SG collected sequences from databases and carried out structure/function analysis of viral proteins. VP developed the ISM software for bioinformatics analysis of viral proteins. HK participated in design of study, interpretation of data and preparation of the manuscript.
